# Assessing healthcare professional wellness during natural and man-made mass casualty incidents: a retrospective study

**DOI:** 10.3389/fpubh.2026.1728587

**Published:** 2026-02-20

**Authors:** Nadav Oppenheim, Uri Aizik, Joseph Offenbacher, Aya Cohen, Michael Markovits, Noaa Shopen, Orna Tal, Gal Pachys, Daniel Trotzky

**Affiliations:** 1 Shamir Medical Center Assaf Harofeh, Tzrifin, Israel; 2 Faculty of Medical and Health Sciences, Tel Aviv University, Tel Aviv, Israel; 3 Department of Emergency Medicine, New York University Grossman School of Medicine, New York, NY, United States; 4 Department of Emergency Medicine, Tel Aviv Sourasky University Medical Center (Ichilov) affiliated to the Gray Faculty of Medical and Health Sciences, Tel Aviv University, Tel Aviv, Israel; 5 Department of Management, Medical Management Program, Bar-Ilan University, Ramat-Gan, Israel; 6 Division of Emergency Medicine, Shaare Zedek Medical Center, Jerusalem, Israel; 7 Hebrew University of Jerusalem School of Medicine, Jerusalem, Israel; 8 Medical Management, Tel Aviv Sourasky University Medical Center (Ichilov) affiliated to the Gray Faculty of Medical and Health Sciences, Tel Aviv University, Tel Aviv, Israel; 9 Department of Epidemiology and Preventive Medicine, School of Public Health, Gray Faculty of Medical and Health Sciences, Tel Aviv University, Tel Aviv, Israel

**Keywords:** burnout, disaster management, mass casualty incident, resilience, secondary traumatic stress

## Abstract

**Introduction:**

Mass Casualty Incidents (MCIs) represent a unique challenge to both healthcare systems and staff, however, limited quantitative research exists to describe the variable impact of natural and manmade MCIs on healthcare professional wellness (HCPW) measures.

**Study objective:**

To compare resilience, burnout and secondary traumatic stress (STS) outcomes between health care professional (HCP) respondents during the Covid-19 (C-19) pandemic and post October 7th War (MM23). We considered factors such as direct patient contact (DPC) and the role of non-clinical activities and previous experiences on HCPW measures.

**Methods:**

A retrospective cross-sectional survey-based study. Continuous variables were summarized as means and standard deviations with analysis including Mann–Whitney U and Kruskal–Wallis tests. Categorical variables were assessed via Chi-square or Fisher’s exact test.

**Results:**

We found no statistically significant difference in resilience between the cohorts (C-19 7.17 vs. MM23 7.01, *p* < 0.112). In contrast, there were significantly higher levels of burnout (C-19 3.59 vs. MM23 3.36, *p* < 0.010) in the C-19 cohort with higher levels of STS (C-19 3.13 vs. MM23 3.36, *p* < 0.020) in the MM23 cohort. When compared to those HCP who did not participate in DPC, those who did engage in DPC in the MM23 cohort reported higher levels of both resilience (DPC 7.21 vs. No DPC 6.8, *p* < 0.001) and burnout (DPC 3.6 vs. No DPC 3.11, *p* < 0.021).

**Conclusion:**

In our retrospective study we found no difference in rates of HCP resilience but did find variable differences in rates of burnout and STS based on the type of MCI. An analysis, considering the association between DPC and HCPW metrics, demonstrated significant differences in the rates of resilience, burnout and STS between the two cohorts. Furthermore, subgroup analysis demonstrated that factors such as non-clinical military service, volunteerism, previous MCI experience and a history of prior military service were all associated with higher levels of respondent resilience during a manmade MCI. These findings demonstrate that differences in HCPW experiences may exist between varying types of MCIs, and that some individual-based, and interpersonal, variables may be associated with differing rates of impact.

## Introduction

Mass-casualty Incidents (MCIs) represent a unique challenge to both healthcare systems and staff. During these high-intensity events, the ability of health care professionals (HCP) to maintain equanimity is essential for optimizing performance and clinical outcomes (1, 2). Consequently, the importance of developing better, evidence-based, approaches to understanding how HCPs experience and react to MCIs has been recognized throughout the medical community. Ultimately, these factors are thought to reflect not only on the professional and psychological competence of the staff, but may also help to predict organizational performance, quality of care metrics, and patient outcomes (3).

The literature addressing the emotional impact of MCIs, and other workplace traumas, on healthcare professional wellness (HCPW) focuses on three distinct, but interrelated, metrics including resilience, burnout and secondary traumatic stress (STS) (3–6). Resilience is commonly defined as adaptability in the face of stress or trauma encountered in the workplace (7). In contrast, burnout is characterized by direct emotional exhaustion, depersonalization, and a reduced sense of personal accomplishment, while secondary traumatic stress refers to more indirect emotional challenges that result from helping or wanting to help a traumatized or suffering person (8, 9). The uniqueness of MCI imposes greater uncertainty and chaos emphasizing the gap between overwhelming need of action and the perceived ability in practice. Still the gap may be influenced by the type of disaster, the number and severity of the casualties, the available resources and personal characteristics of the therapists. Limited research, however, exists to assess the association between different types of MCI events and HCP responses to these important wellness measures. Furthermore, there remains a lack of evidence-based studies to describe how both work-based and non-work-based factors may be associated with variability in HCPW metrics during these events.

In the wake of the COVID-19 pandemic (C-19) we conducted, and published, a comprehensive retrospective study evaluating conditions of resilience, burnout and STS on HCP respondents at a tertiary academic hospital utilizing the validated: Connor-Davidson resilience scale (CD-RISC-10), Maslach burnout inventory (MBI), and Professional quality of life scale (ProQOL) (10). Nearly 3 years after that event, on October 7, 2023, our hospital was again confronted with a large MCI following the October 7th massacre and ensuing Israel-Hamas War (MM23) (11). To better understand the impact of these events on our healthcare personnel, we again conducted a retrospective survey incorporating the CD-RISC-10, MBI and ProQOL utilizing the same assessment tool as for the C-19 cohort.

In recognizing that these events enabled us to study the variable challenges faced by HCPs during both natural and man-made MCIs, our primary analysis and study outcome looked to compare resilience, burnout and STS between HCP respondents in the C-19 and MM23 cohorts. Our secondary analysis, and subsequent secondary study outcome sought to evaluate the association between different levels of direct patient contact (DPC) and HCPW measures between respondents in the C-19 and MM23 cohorts. As a subgroup analysis of our study’s secondary analysis, we considered the association of ongoing, non-clinical, activities (including both reserve military conscription and non-clinical volunteerism) as well as previous adaptive-capacity building experiences (including prior military service and experiences with MCIs) on our HCPW metrics.

We hypothesized that factors related to the direct personal threats to HCP during the C-19 pandemic as well as the national uncertainty in the MM23 period would variably impact HCPW measures. Additionally, we hypothesized that the association between DPC and HCPW measures would be vary based on the type of MCI event. Ultimately, the aim of this study was to describe the variability of the association of different MCI events on our healthcare workforce wellness to provide a more comprehensive, evidence-based model to contribute to future research and clinical preparedness.

## Materials and methods

We conducted a retrospective cross-sectional survey-based study to assess levels of resilience, burnout and STS among HCP working in a tertiary, academic public hospital in central Israel. Data was collected between May 2024 and November 2024, during the MM23 period. The hospital has approximately 900 beds and provides medical services to over 1 million residents in the central region of the country. The emergency department of this hospital is among the largest in Israel, treating over 160,000 patients annually (12).

The survey consisted of 33 questions assessing resilience, burnout and STS. Resilience was measured using the 10-item CD-RISC-10. Responses were rated on a 10-point Likert scale and averaged to a total score, with higher scores indicating greater resilience. Burnout was assessed using an adapted version of the MBI, incorporating 13 components rated on a 10-point scale. Higher total scores reflected more severe burnout symptoms. STS was evaluated using a modified ProQOL scale with 10 items focusing on secondary trauma. Responses were scored from 1 (never) to 10 (very often), with higher scores indicating greater levels of secondary traumatic stress.

347 participants from various hospital departments took part in the MM23 study cohort. All participants were active staff members who voluntarily completed an anonymous questionnaire distributed electronically via institutional email and Short Message Service (SMS) messenger. The research protocol was reviewed and approved by the hospital’s Helsinki Committee acting as the Independent Review Board (IRB), and all procedures conformed to ethical standards.

REDCap (Research Electronic Data Capture) was used for data collection and management. Descriptive statistics were used to report sample characteristics. Categorical variables were summarized as frequencies and percentages which were calculated based on the total number of respondents for each individual question. Continuous variables were summarized as means and standard deviations. Normality of continuous variables was evaluated using histograms and Q–Q plots. Comparisons of continuous variables between two groups were performed using the Mann–Whitney U test, and comparisons between more than two groups were performed using the Kruskal–Wallis test. Comparisons of categorical variables were conducted using the Chi-square test or Fisher’s exact test when appropriate. All statistical tests were two-tailed, and a *p*-value < 0.05 was considered statistically significant. Statistical analyses were performed using IBM SPSS Statistics for Windows, Version 25 (IBM Corp., Armonk, NY, United States). Cronbach’s alpha was calculated to assess the internal consistency of the survey instruments.

## Measurement tools

### The Connor-Davidson resilience scale (CD-RISC-10)

We utilized the CD-RISC 10-itemScores in Non-treatment Seeking Trauma Survivors to measure four components of resilience: optimism, meaningfulness/purpose, resourcefulness/self-efficacy, and hardiness. Each question had five answer choices: not true at all (0), rarely true (1), sometimes true (2), usually true (3), mostly true (4), and true nearly all of the time (e.g., “always true”) (5). In addition, the total scoring scale was modified to be scored 1–10, with higher scores indicating higher resilience.

### Maslach burnout inventory

An adoptive version containing 13 of the 16 components was utilized with a 10-point scale The higher the total score, the more severe the burnout.

### Professional quality of life scale

Professional Quality of Life Scale (ProQOL) was utilized to measure secondary trauma, was with a 1 to 10 answer scale: 1 indicated that the participant never felt this emotion and 10 if the emotion was felt very often. Thus, the lower the ProQOL score, the lower the secondary trauma experienced. To assess secondary trauma, we used the validated official Hebrew ProQOL questionnaire 4th edition adapted modified version of 10 out of the 30 questions was used; those 10 questions focused on secondary traumatization (10).

## Results

Our primary MM23 study cohort consisted of 347 total survey respondents. Of those 235 (68.3%) were women, 69 (21.2%) were physicians, 141 (43.3%) were nurses, 92 (43.6%) worked in the emergency department, and 117 (34.0%) held management roles at the departmental or institutional levels. 176 (51.1%) reported engaging in DPC with casualties of the October 7^th^ massacre and subsequent Israel-Hamas War. Outside of their HCP duties 36 (10.5%) of the respondents were drafted into reserve military service and 115 (33.5%) participated in some form of volunteer service. Regarding experiences prior to the conflict 122 (35.7%) respondents reported having previously experienced an MCI while 175 (51.0%) reported prior military experience through national service (Table 1).

**Table 1:** Distribution of resilience, traumatization and burnout scores by population characteristics in the MM23 cohort.

Category	Characteristics	Number of respondents	Resilience (measured by CD)	Traumatization (measured by ProQOL)	Burnout (measured by MBI)
	Total population	347	7.01	3.36	3.36
Gender	Man (31.7%)	109	7.28	3.2	3.57
	Woman (68.3%)	235	6.88	3.44	3.27
Age	Below 40 (42.4%)	146	7.05	3.22	3.57
	Over 40 (57.6%)	198	6.98	3.47	3.22
Religion	Jewish (86.0%)	295	7.00	3.26	3.18
	Non-Jewish (14.0%)	48	6.99	4.00	3.69
Profession	Physician (21.2%)	69	7.34	3.57	3.51
	Nurse (43.3%)	141	9.97	3.28	3.74
	Allied Health workers (16.9%)	55	7.02	3.34	2.62
	Administration (18.7%)	61	6.93	3.44	2.98
Department	ED (43.6%)	92	7.57	3.21	3.98
	Inpatient (44.5%)	94	6.84	3.57	3.35
	Ambulatory (11.9%)	25	7.18	3.88	3.34
Management	No (66.0%)	227	6.86	3.48	3.66
	Yes (34.0%)	117	7.08	3.30	3.21

**Figure 1:**
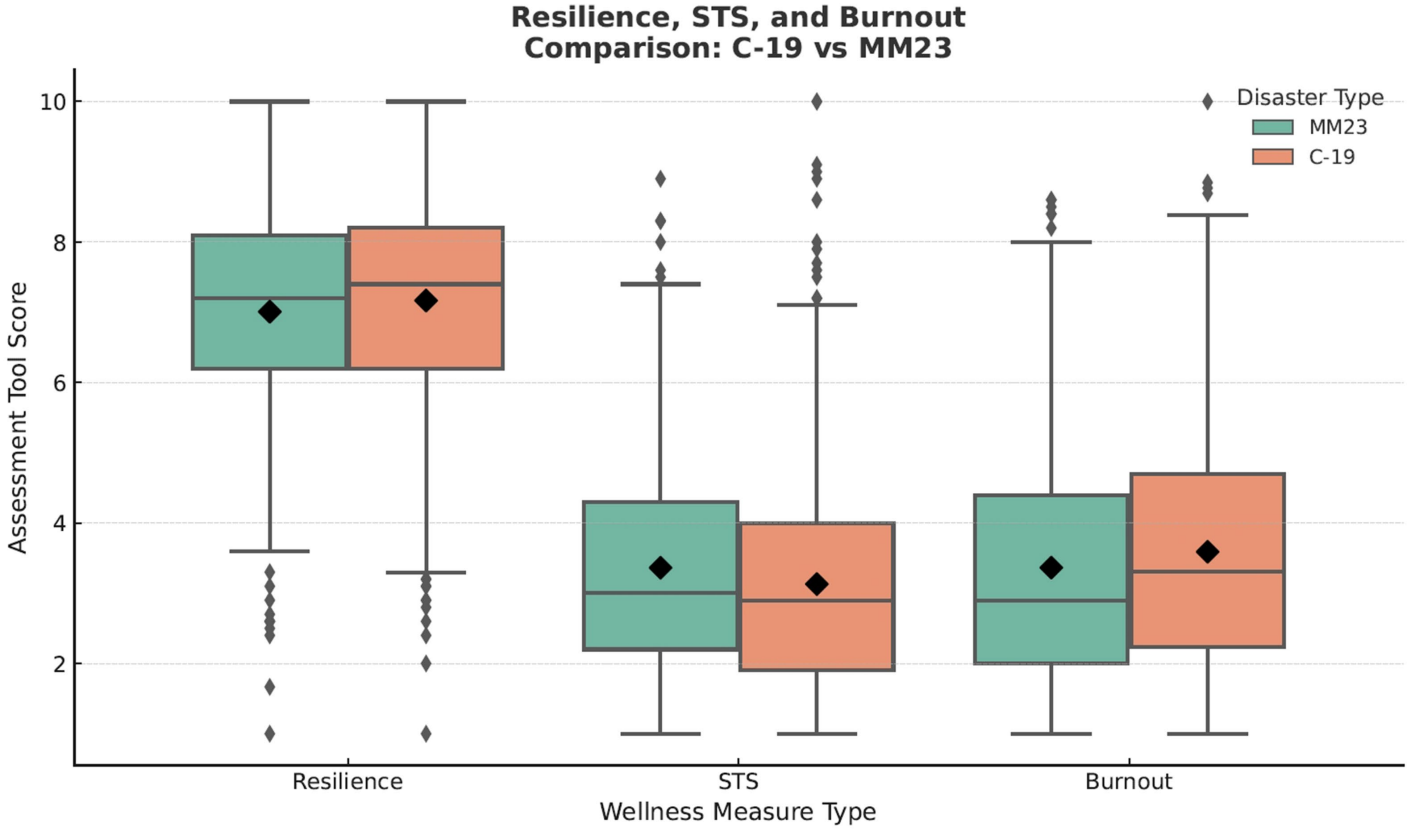
Scores for resilience reflect the CD-RISC tool. STS reflects the secondary traumatic stress (ProQOL subscale), and Burnout reflects the MBI questionnaire.

For our primary analysis we compared our MM23 study group to a previously studied C-19 cohort consisting of 655 respondents at the same tertiary healthcare center (10) with a primary outcome assessing differences in mean rates of resilience, burnout and STS between the two cohorts. We found no statistically significant difference in resilience scores between the two cohorts (C-19 7.17 vs. MM23 7.01, *p* = 0.112). In contrast, there were significantly higher levels of burnout (C-19 3.59 vs. MM23 3.36, *p* = 0.010) in the C-19 cohort with higher levels of STS (C-19 3.13 vs. MM23 3.36, *p* = 0.020) in the MM23 cohort (Table 2; Figure 1).

**Table 2:** Distribution of resilience, traumatization and burnout scores by disaster type cohort.

Category	Characteristics	Number of respondents	Resilience (measured by CD)		Traumatization (measured by ProQOL)		Burnout (measured by MBI)	
Disaster type	C-19 (65.4%)	655	7.17	*p* = 0.112	3.13	*p* = 0.020	3.59	*p* = 0.010
	MM23 (34.6%)	347	7.01		3.36		3.36	

Our secondary analysis sought to evaluate the impact of DPC on respondents between the MM23 cohort and the C-19 cohort. When compared to those HCPs who did not participate in DPC, those who did engage in DPC in the MM23 cohort reported statistically significant higher levels of both resilience (DPC 7.21 vs. No DPC 6.8, *p* < 0.001) and burnout (DPC 3.6 vs. No DPC 3.11, *p* = 0.021). No significant difference was found in rates of STS between the two subgroups (DPC 3.34 vs. No DPC 3.39, *p* = 0.905).

Findings from the C-19 cohort demonstrated that, as opposed to those HCP who did not participate in DPC, those who engaged in DPC reported significantly lower levels of resilience (DPC 7.01 vs. No DPC 7.44, *p* = 0.002), higher levels of burnout (DPC 3.85 vs. No DPC 3.14, *p* < 0.001) and higher levels of STS (DPC 3.28 vs. No DPC 2.86, *p* < 0.001; Table 3).

**Table 3:** Distribution of resilience traumatization and burnout scores by direct patient contact (DPC) and disaster type.

Characteristics	Number of participants	Resilience (measured by CD) sig		Traumatization (measured by ProQOL) sig		Burnout (measured by MBI) sig	
C-19: No DPC (36.3%)	236	7.44	*p*= 0.002	2.86	*p* < 0.001	3.14	*p* < 0.001
COVID: Yes DPC (63.7%)	415	7.01		3.28		3.85	
MM23: No DPC (49.0%)	169	6.80	*p* = 0.001	3.39	*p* = 0.905	3.11	*p* = 0.021
MM23: Yes DPC (51.0%)	176	7.21		3.34		3.60	

To assess for additional variables that might be associated with differences in HCPW metrics we conducted a subgroup evaluation of our secondary analysis looking to assess the possible role of non-clinical activities (including both reserve military conscription and non-clinical volunteerism) as well as previous experiences (including prior military service and experiences with MCIs) on resilience, burnout and STS metrics among respondents in the MM23 cohort.

Regarding non-clinical, activities during the ongoing conflict, respondents who reported participation in compulsory reserve military service (RMS) during the ongoing conflict reported significantly higher levels of resilience (RMS7.52 vs. No RMS 6.95, *p* = 0.002) and without any significant differences in either burnout (RMS 3.37 vs. No RMS 3.36, *p* = 0.64) or STS (NMS 3.01 vs. No NMS 3.40, *p* = 0.11). For respondents who reported engagement in non-clinical volunteerism we found statistically higher levels of resilience (Volunteer 7.26 vs. No Volunteer 6.89 *p* = 0.02) and higher levels of traumatization (Volunteer 3.55 vs. No Volunteer 3.26 *p* = 0.05). There was no difference in reported levels of burnout (Volunteer 3.15 vs. No Volunteer 3.47 *p* = 0.23).

Regarding the association between HCPW metrics and previous adaptive-capacity building experiences, respondents who reported prior MCI (PMCI) encounters also reported significantly higher levels of resilience (PMCI 7.28 vs. No PMCI 6.86, *p* < 0.001) without any significant differences in either burnout (PMCI 3.45 vs. No PMCI 3.32, *p* = 0.62) or STS (PMCI 3.33 vs. No PMCI 3.37, *p* = 0.57). In addition, we found that those who had a history of prior engagement in national military service (NMS) reported significantly higher levels of resilience (NMS7.21 vs. No NMS 6.79, *p* = 0.010) and lower levels of both burnout (NMS 3.17 vs. No NMS 3.57, *p* = 0.021) and STS (NMS 3.09 vs. No NMS 3.65, *p* < 0.001; Table 4).

**Table 4:** Distribution of resilience traumatization and burnout scores by personal activity among the MM23 cohort.

Category	Response	Number of respondent	Resilience (measured by CD)		Traumatization (measured by proqol)		Burnout (measured by MBI)	
Prior military service	No (49.0%)	168	6.79	*p* = 0.010	3.65	*p* = 0.001	3.57	*p* = 0.021
	Yes (51.0%)	175	7.21		3.09		3.17	
Reserve military service	No (89.5%)	307	6.95	*p* = 0.002	3.4	*p* = 0.108	3.36	*p* = 0.638
	Yes (10.5%)	36	7.52		3.01		3.37	
Volunteer activity	No (66.5%)	228	6.89	*p* = 0.022	3.26	*p* = 0.047	3.47	*p* = 0.232
	Yes (33.5%)	115	7.26		3.55		3.15	
Past MCI experience	No (64.3%)	220	6.86	*p* = 0.001	3.37	*p* = 0.571	3.32	*p* = 0.618
	Yes (35.7%)	122	7.28		3.33		3.45	

## Discussion

In the wake of the COVID-19 Pandemic and ongoing conflicts throughout the world, the international medical community has developed a stronger recognition of the importance of understanding factors that impact HCPW. Understanding that factors such as resilience, burnout and secondary traumatic stress can deeply impact individual and systems-based practice and performance, increased efforts are actively being made to support and strengthen the healthcare workforce (13, 14). To contribute to this developing area of study we conducted a series of retrospective survey-based studies in the wake of the C-19 pandemic and MM23 massacre to contribute to an evidence-based understanding of how these different MCI events impacted our hospital’s healthcare workforce.

The primary outcome of our study looked to compare the varying impact of the C-19, natural MCI, to the MM23, man-made MCI, on previously established metrics used to describe HCPW. We found that both types of MCI events were associated with a similar reporting of resilience between both cohorts. Burnout (a measure of emotional exhaustion, depersonalization, and reduced accomplishment in the workplace) (8) was found to be significantly higher among members of the C-19 cohort, whereas STS (a measure of vicarious trauma symptoms arising from caring for the traumatized patient) was significantly higher among members of the MM23 cohort (9).

These findings are consistent with the existing literature demonstrating that burnout measures are mainly driven by chronic workload, uncertainty and shortages of both supplies and manpower (8). These features were recognized as foundational characteristics of the challenges faced by HCW during the C-19 pandemic (15, 16). In contrast, STS captures trauma-like symptoms that arise from a closeness of proximity to those who are ill and injured (9). This metric is generally accepted as a measure of an indirect experience, as the HCW generally does not personally experience a direct threat to one’s own health and safety secondary to their clinical work (17, 18). These features came to define the MM23 experience for healthcare workers caring for the injured of both the massacre and subsequent war. In the context of our two studied cohorts, the system-wide pressure and direct infection risk of C-19 likely contributed to increased rates of burnout, while the intense and morally shocking nature of MM23 experience amplified STS of those caring for the victims.

Another important finding of our primary analysis demonstrated similarity in resilience measures responses between the two studied cohorts further sporting the existing literature which suggest that resilience may function as a source of stability and consistency during times of crisis (15). An important feature of resilience, as opposed to the other studied characteristics, is that it is highly modifiable based on individual and interpersonal factors such as resilience-specific training, disaster preparedness exercises and post event debriefing (16).

As both burnout and STS are often correlated with proximity to both direct personal threats and secondary exposure to the sick and injured (8, 9), the primary objective of our secondary analysis sought to assess how DPC, mainly shouldered by frontline HCPs, was variably associated with differences in resilience, burnout and STS metrics between the two study cohorts. During C-19, DPC was associated with lower resilience and higher levels of burnout and STS compared with non-DPC roles. This pattern aligns with evidence that frontline clinicians in pandemics face persistent infection risks, heavy workload, frequent changes in protocols and reduce accesses to usual support systems, such as colleagues, family and friends due to the isolation and infection control measures. Furthermore, these factors undermine coping capacity and cause exhaustion and STS (19–21).

In contrast, following the MM23 MCI, DPC was linked with higher resilience, only a small increase in burnout and no clear difference in STS relative to non-DPC HCPs. In a secured hospitable setting, where frontline roles did not add additional personal medical risk, direct involvement and meaningful connections foster resilience by enhancing professional fulfillment, purpose, and a sense of connection (22–24). High emotional exposure particularly in trauma care, however, demonstrated increased risk of burnout and secondary traumatic stress (1). Our findings ultimately support the notion that the impact of DPC on HCPW is highly dependent on the context of the event itself. In a natural MCI, such as the C-19 pandemic DPC often adds to constant personal danger and wears them down. While man-made MCI such as terror related emergencies, can strengthen resilience by giving a sense of mission without extra personal risk.

Additionally, as a secondary outcome measure of a subgroup of our secondary analysis we focused exclusively on our study’s primary MM23 cohort to assess the possible association of non-clinical, activities (including both reserve military conscription and non-clinical volunteerism) and previous personal experiences (including prior military service and experiences with MCIs) on our resilience, burnout and STS metrics. While these findings demonstrated variable impacts on both STS and burnout measures, the literature indicates that these association are context dependent: prior or reserve military service has been linked to greater trauma exposure and higher odds of screening positive for stress disorders (25). By contrast nonclinical volunteerism during crises has been associated with lower burnout and may buffer the negative impact (26). Based on literature, evidence is currently limited, and prior trauma exposure generally raises the risk of later PTSD and STS among emergency personnel and health workers (27, 28). In our MM23 subgroup, some non-clinical involvement lined up with higher resilience, with only small shifts in burnout and STS. The effect is not the same across roles, it depends on role demands, the support people have, and the work environment. Secured, well-supported settings tend to align with resilience, heavier demands or limited support tilt toward burnout and STS.

## Limitations

Our study was a retrospective, self-reported, survey and as such findings may be compromised by several biases including recall and selection bias. Despite both studies being conducted in the same medical institution, differences between the two study groups are noted to exist, including, differences in survey response rates. Additionally, differences in survey and event periods may contribute to findings. As these surveys were conducted anonymously, it was not possible to identify which, if any, respondents spanned both study cohorts.

The study was conducted at a single clinical site, and as a result, findings may not be generalizable across all healthcare environments. Furthermore, although the two events being studied do fall into broad classifications of natural and man-made MCIs unique features of the COVID-19 pandemic and the MM23 experiences may have limited the generalizability of results.

## Conclusion

In our retrospective study of two cohorts of HCWs, from a tertiary medical institution, that experienced both a natural and manmade MCI we found no difference in rates of HCW resilience but did find variable differences in rates of burnout and STS based on the type of MCI experienced. A secondary analysis, considering the association between DPC and HCPW metrics, demonstrated significant differences in the rates of resilience, burnout and STS between the two cohorts in line with current literature pertaining to these wellness measures. Furthermore, subgroup analysis demonstrated that factors such as non-clinical military service, volunteerism, previous MCI experience and a history of prior military service were all associated with higher levels of respondent resilience during a manmade MCI event. These findings demonstrate that differences in HCPW experiences may exist between varying types of MCIs, and that some individual-based, and interpersonal, variables may be associated with differing rates of impact.

## Data Availability

The original contributions presented in the study are included in the article/supplementary material, further inquiries can be directed to the corresponding author.
